# Using Virtual Reality to Assess and Promote Transfer of Memory Training in Older Adults With Memory Complaints: A Randomized Controlled Trial

**DOI:** 10.3389/fpsyg.2021.627242

**Published:** 2021-03-12

**Authors:** Benjamin Boller, Émilie Ouellet, Sylvie Belleville

**Affiliations:** ^1^Department of Psychology, Université du Québec à Trois-Rivières, Trois-Rivières, QC, Canada; ^2^Research Centre, Institut Universitaire de Gériatrie de Montréal, Montréal, QC, Canada; ^3^Department of Psychology, Université de Montréal, Montréal, QC, Canada

**Keywords:** cognitive training, episodic memory, virtual reality, aging, memory complaint, randomized controlled trial

## Abstract

In this proof-of-concept study, we assessed the potential for immersive virtual reality (VR) to measure transfer following strategic memory training, and whether efficacy and transfer are increased when training is complemented by practice in an immersive virtual environment. Forty older adults with subjective memory complaints were trained with the method of loci. They were randomized to either a condition where they practiced the strategy in VR (*n* = 20) or a control condition where they were familiarized with VR using a non-memory task (*n* = 20). Training efficacy was measured with word recall, and transfer of the training benefit was measured with a recall task completed in two VR tasks (primary outcomes) as well as a self-report memory questionnaire (secondary outcomes). Testing was administered before (PRE), midway (POST 3), and after (POST 6) training. Participants improved their scores on word recall. Regarding transfer measures, participants improved their performance in the two VR recall tasks but not on the self-report memory questionnaire. No significant group effect was observed. Improvement was found when comparing PRE to POST 3 with no further improvement at POST 6. Thus, strategic memory training improved the memory of seniors with memory complaints on word recall and a transfer task relying on a VR scenario that resembles real-life. However, no evidence supporting an increase in transfer effects was found when enriching training with VR memory exercises.

## Introduction

One of the primary concerns for older adults is age-related memory loss, which is targeted by cognitive intervention programs to reduce the impact of cognitive decline. Memory training generally involves teaching mnemonics known to improve the quality of encoding and that facilitate later retrieval (Montreal Cognitive Assessment, MoCA; Willis and Belleville, 2016). Strategic memory training is found to increase memory performance in healthy seniors (e.g., Ball et al., 2002; Engvig et al., 2010) as well as those with mild cognitive impairment (MCI; Akhtar et al., 2006; Belleville et al., 2006, 2018; Hampstead et al., 2008; Kinsella et al., 2009; Lim et al., 2012; Olchik et al., 2013). The finding that older adults remain capable of learning new memory strategies has advanced the field, since it demonstrates that cognition and the brain remain plastic even in old age. Yet, the ultimate goal of cognitive training is not to improve memory for words learned in experimental contexts, but to transfer and improve memory in everyday life. Transfer refers to the notion that training one cognitive function, or one set of materials, will improve performance on untrained functions or materials (Butterfield and Nelson, 1991; Perkins and Salomon, 1992; Mayer and Wittrock, 1996; Austin, 2009; Noack et al., 2009). There are different types of transfer: *Content transfer* refers to improvement of cognitive abilities or tasks that use cognitive processes that are similar to those that were trained. For instance, content transfer may be measured by examining whether learning to memorize visual material results in improved learning of auditory material. In turn, *context transfer* occurs when a behavior or strategy learned in one context is successfully applied or leads to improvement in a different context (Salomon and Perkins, 1988; Perkins and Salomon, 1992; Bransford et al., 2000; Lobato, 2006). In health studies, transferring training effects from the clinic or laboratory to activities in real-life is one of the most sought-after *context transfers*. However, generating *context transfer* to real-life following cognitive training is challenging (for reviews, see Rebok et al., 2007; Zelinski, 2009; Simons et al., 2016). The lack of clear evidence for transfer in real-life has been raised as one of the major limitations for recommending cognitive training as a tool to reduce the impact of cognitive decline in older adults (for a statement, see Max Planck Institute for Human Development and Stanford Center on Longevity, 2014).

One major challenge is the difficulty of accurately measuring *context transfer*. Most past studies measured *context transfer* with self-report questionnaires, where participants were asked to judge their memory capacities in different everyday life situations. Measuring transfer with a self-report questionnaire leads to inconsistent findings. For example, a recent randomized controlled study compared a strategy-based memory training condition to a psychosocial intervention and a no-contact control condition in older adults with MCI. The study did not observe transfer on self-report measures of complex activities of daily living. However, participants reported an increased use of memory strategies in everyday life (Belleville et al., 2018). In the ACTIVE randomized controlled trial, memory-trained participants reported less difficulty performing daily activities compared to controls, but only at the 10-year follow-up (Ball et al., 2002; Willis et al., 2006; Rebok et al., 2014). Finally, a recent meta-analysis reported positive but small effects of cognitive training on activities of daily life in activity questionnaires, as well as on metacognitive measures in older adults with MCI (Chandler et al., 2016). Self-report measures require good metacognitive abilities and are easily influenced by variables such as mood or personality. These limitations may account in part for small and inconsistent effects that are reported in the literature. This justifies the need to develop different measures to evaluate *context transfer* effects.

Another possible explanation for the limited evidence for *context transfer* relates to how training programs are delivered. Transfer is facilitated when there are similarities between the learning experience and the context where training will be applied. However, training is usually delivered through classroom-based and instructor-centered exercises, performed in the laboratory or with computer exercises, which differ widely from everyday life conditions. This can increase the challenge for seniors when it comes to applying the strategies. When placed in an environment different from where they were trained, learners may not recognize appropriate situations to apply the strategies or may perceive them as inapplicable or inappropriate (Elangovan and Karakowsky, 1999). Therefore, offering exercises in a context close to the environment where the learner will apply strategies is expected to promote transfer (Elangovan and Karakowsky, 1999; Yorks et al., 2003). Some studies have attempted to encourage transfer by using verbal exercises containing scripts of practical situations (Jobe et al., 2001; Ball et al., 2002; Rapp et al., 2002; Belleville et al., 2006; Willis et al., 2006; Troyer et al., 2008; Brum et al., 2009), but several have failed to find evidence for *context transfer* (Ball et al., 2002; Rapp et al., 2002; Willis et al., 2006; Troyer et al., 2008). This could be because these scripts were provided in classroom environments, thus remaining artificial and relatively removed from complex real-life memory tasks.

Virtual reality (VR) has the potential to measure and promote *context transfer*. VR is a computer-based technology that allows users to interact with a multisensory simulated environment in real time (Saposnik and Levin, 2011). VR allows the creation of environments and tasks that mimic real life situations (Rizzo et al., 2004). Several studies have demonstrated its feasibility (Rose et al., 2005; Shuchat et al., 2012; Lecavalier et al., 2020) and validity to measure cognition (Gould et al., 2007; Jiang and Li, 2007; Plancher et al., 2010; Gonneaud et al., 2014; Jebara et al., 2014; Ouellet et al., 2018; Lecavalier et al., 2020), as well as its ecological validity to reflect real-life cognition (Waller et al., 2001; Zhang et al., 2003; Cushman et al., 2008; Widmann et al., 2012; Sorita et al., 2013; Allain et al., 2014; Parsons, 2015; Ouellet et al., 2018). VR can be used to develop complex training environments that closely resemble the real-world, while allowing precise control of stimulus presentation and response collection (Rizzo and Buckwalter, 1997).

A few cognitive training studies have relied on VR and reported beneficial impacts on real-life measures. For example, participants who trained their shopping skills in a virtual supermarket were found to improve these skills in real-life situations (Cromby et al., 1996; Yip and Man, 2013). Training in a virtual replica of a hospital (Brooks et al., 1999) or a city district (Wallet et al., 2009, 2013) improved route navigation abilities in the corresponding real environments. These results suggest that providing training in a virtual environment can transfer to similar real-life situations. However, none of these studies have explored whether adding VR exercises to a cognitive intervention, which was previously shown to improve cognitive performance, can increase transfer effects.

This study had three main goals: (1) to assess *context transfer* using VR and compare it to self-report questionnaires; (2) to test whether including VR in training can increase *context transfer* effects by providing training conditions that are close to real life situations; and (3) to assess if efficacy and *context transfer* vary with the number of training sessions (Lampit et al., 2014; Schwaighofer et al., 2015), as it is possible that transfer requires a larger training dose for an observed effect than what is suggested by efficacy measures.

All participants received a classroom-based memory strategy training with the method of loci. Half of the participants were randomized to an experimental condition, where they practiced memory exercises in the *Virtual Shop*, a fully immersive 3D VR convenience store. The other half of participants were randomized to an active control group and received memory training but did not practice memory exercises in VR. Performance on a classical word-memory task provided information on training efficacy. Context transfer was measured with a VR task, where participants memorized and retrieved an errand list in a Virtual Shop (Ouellet et al., 2018; Lecavalier et al., 2020). The errand list was similar to the one received in training in a VR task, where they learned words while finding directions in a Virtual Car Ride (Bier et al., 2018). They also answered a self-report questionnaire, where they rated their everyday memory ability, to compare VR-based transfer with more traditional self-report measures. We assessed efficacy and transfer halfway through training and upon completion of training to examine how efficacy and transfer developed with additional training sessions (Lampit et al., 2014; Schwaighofer et al., 2015). This design will help assess whether insufficient dosage may account for the lack of solid transfer effects in previous studies.

## Materials and Methods

### Participants

Participants were independent community-dwelling older adults with subjective cognitive decline (SCD), who were recruited through advertisements in community centers, public conferences and magazines for seniors. Participants included in the study complained about their memory but were cognitively intact, which is consistent with current SCD criteria (Jessen et al., 2014). Presence of a memory complaint was identified by asking participants if they believed their memory was worse than it used to be. Participants were asked to complete a series of neuropsychological tests to determine whether they were cognitively intact (see below). Other inclusion criteria were being over age 50 and fluent in French, and having normal or corrected vision and hearing. Participants were excluded if they reported balance problems, substance abuse, presence or history of a neurological disorder, stroke or severe traumatic brain injury, presence or history of a severe psychiatric disorder (e.g., schizophrenia, post-traumatic stress disorder, and recurrent episodes of major depression), fibromyalgia, uncontrolled sleep apnea, fatal disease (e.g., cancer), general anesthesia in the past 6 months, and a diagnosis of MCI or dementia.

An initial phone interview was used to assess the presence of a memory complaint and screen for exclusion criteria. Eligible participants were invited to complete a more thorough clinical and neuropsychological assessment to confirm that they met inclusion criteria and determine their characteristics. Cognitive tests used to determine normal cognition included the MoCA (Nasreddine et al., 2005), two tests for episodic memory; the 16 item Free and Cue Recall (RL/RI-16; Van der Linden et al., 2004) and the French version of the Logical Memory I subtest from the Wechsler Memory Scale III (LM I; Wechsler, 1997), a language test: the Boston Naming Test (BNT; Kaplan et al., 2001), a test assessing executive function: the French version of the Stroop-Victoria test (Troyer et al., 2006; Moroni and Bayard, 2009; Tremblay et al., 2016), and a test evaluating crystallized intelligence: the Vocabulary subtest from the Wechsler Adult Intelligence Scale IV (Wechsler, 2008). The cut-off for a normal score on the MoCA was ≥26 (Nasreddine et al., 2005). Performance on the RL/RI-16, BNT, Stroop-Victoria, and vocabulary tests were deemed normal when scores were no more than 1.5 SDs below the mean of age- and education-matched normative samples. The score on LM I was considered normal based on education-adjusted cut-off scores used in the Alzheimer’s Disease Neuroimaging Initiative study (ADNI; Bennett et al., 2002). Clinical tests used for characterization included the Geriatric Depression Scale (GDS; Yesavage et al., 1982), the Hachinski Scale (Hachinski et al., 1975), the Charlson Comorbidity Index (Charlson et al., 1987), and the Activities of Daily Living-Prevention Instrument (ADL-PI, from ADCS; Galasko et al., 2006).

The sample size was determined from data of a previous memory training study (Belleville et al., 2006) and based on a power analysis with g*power software version 3.1.9.7. It was estimated that 20 participants per intervention arm would provide 80% power to detect a large effect size difference in the mean change from PRE to POST 6 in verbal recall scores (paired-sample *t*-test with a 5% 2-sided significance level). After allowing for an estimated dropout rate of 15%, we required 48 patients to be randomized.

### Design

**Figure 1:**
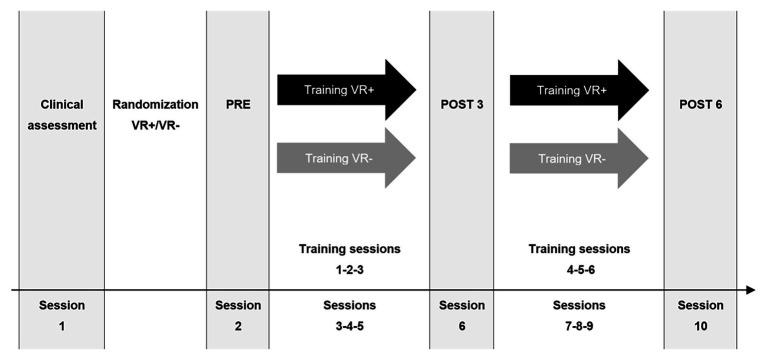
Study design. The figure shows the design from session 1 to 10 in the study. Black (VR+) and gray (VR−) arrows represent the differences between the two randomized conditions.

This was a two-arm, double blind, randomized active-controlled trial (parallel group, block randomization, *N* = 8). The protocol adheres to the Consolidated Standards of Reporting Statement for Social and Psychological Interventions (CONSORT-SPI; Grant et al., 2018; Montgomery et al., 2018; see Figure 1). All participants received memory training with the method of loci and completed VR exercises. They also received VR exercises, which differed depending on the condition to which they were randomized: Half of participants were randomized to a condition where they practiced the method of loci in a Virtual Shop (VR+), and the other half were randomized to an active control condition where they were placed in the same virtual environment but only performed visuo-motor exercises (VR−). This condition was considered as an active control and ensured that the improvement in the transfer task found in the VR+ participants was not due to increased familiarity with VR or the response procedure. Randomization was done in waves of eight participants, after they were tested for inclusion/exclusion criteria, but before the baseline (PRE) assessment (see Figure 1). The randomization was performed using a computerized random list (ALEA function of Excel) by a research assistant, who was not involved in the project.

Training occurred over six 1-h sessions, every other weekday over 2 weeks. Participants who missed a session were offered a make-up session. Outcome measures were taken no more than 1 week prior to training (PRE), 1 week after the third training session (POST 3) and no more than 1 week following the end of the training (POST 6).^1^ For each time point, outcome measures were taken during a single session. The order of presentation of the tasks used as outcome measures was similar across participants and sessions. Alternative versions of the memory tasks were used for each time point and randomly allocated to participants. To minimize performance variability due to circadian effects, participants were evaluated at the same time of day (morning or afternoon) for the three outcomes sessions.

The study was double-blind: the examiner was blind to the participant’s training allocation and participants were unaware of the two different VR training conditions. To reduce expectancy, and enforce double-blinding, the consent form mentioned only that the participant would have to *complete exercises in VR that consist in locating and buying products in a shop*, and hence no mention was made of randomization. The therapist involved in teaching the memory strategy was blind to the hypotheses and the participants’ VR conditions. The therapist involved in supervising the VR exercises was not blind to the VR condition.

The study was conducted at the Research Center of the Institut universitaire de gériatrie de Montréal (CRIUGM). The protocol was approved by the Comité mixte d’éthique de la recherche du Regroupement Neuroimagerie/Québec (CMER-RNQ) and all participants completed a written informed consent form prior to study participation.

#### Intervention

All participants were trained with the method of loci strategy, which was provided in small groups of 4–5 participants during weekly three 50-min sessions over 2 weeks. Each session was completed with an individual 10-min VR exercise session (VR+ or VR−).

The method of loci is a well-known mnemonic strategy, which relies on mental imagery and associative memory, where participants mentally associate items with a familiar sequence of loci situated in their environment (Lea, 1975). First, participants identify and learn a set of loci in a familiar route, usually in their house or apartment. They then learn to associate each loci with words or events. At encoding, interactive visual imagery is used to associate the item and its locus in serial order (i.e., the nth word is associated to the nth locus). When the list is recalled, the participant mentally goes through its mental route, retrieving the associated image at each locus (Verhaeghen and Marcoen, 1996). For example, if a participant must remember to buy a list of items, the first two being a lemon and milk, these two items will be associated with the first two loci on their mental route. If the front door of the participant’s apartment is assigned as the first locus, the participant may create an interactive image where the front door handle has been replaced by a lemon. If a vestibule bench is the second loci, the participant can visualize milk flowing from the bench like a waterfall. Subsequently when shopping, the participant will mentally pass through the first locus with the lemon handle, then mentally pass in front of the second locus and see the milk waterfall. The method of loci was used here by adapting the content from the MEMO program (Belleville et al., 2006, 2018). The number of sessions and trials was increased relative to the original MEMO program. Each session lasted about 50 min and included a face-to-face teaching of the method of loci in small groups of 2–4 participants. Session 1 consisted of exercises to improve mental imagery and teach participants to create interactive mental images. Participants were encouraged to create bizarre, distinctive, and/or funny interactive images as these are known to enrich the memory trace and facilitate retrieval. The method of loci was then presented, and participants were asked to memorize a mental route with 12 familiar loci from their home. Session 2 began with a review of the steps involved in the method of loci and the participant’s individual route. The method was then practiced using lists of concrete and imaginable words that were visually presented on a PC computer run by E-prime 2 (Psychology Software Tools, Sharpsburg, PA). The therapist asked the participant to describe their interactive mental images for the first exercises so that guidance and feedback on the quality of the interactive images could be provided. Sessions 3–6 included additional exercises with varying lists of 12 concrete words that participants were instructed to encode and retrieve with the method of loci. Feedback on performance and interactive images continued to be provided. The difficulty level was increased gradually over these sessions by shortening the time allowed to create the interactive images and reducing feedback and guidance.^2^

**Figure 2:**
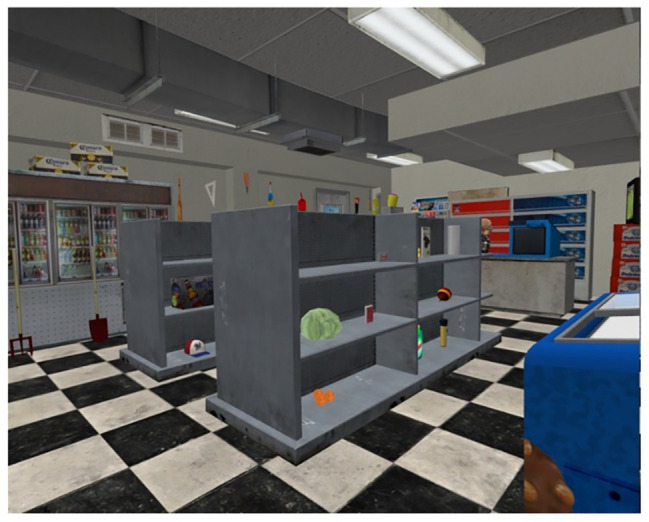
Screen shot captured from the Virtual Shop.

The virtual exercises took place in the Virtual Shop (see Figure 2) environment, which is a three-dimensional and fully immersive virtual convenience store, where participants can walk freely to search for common shopping objects (Ouellet et al., 2018; Lecavalier et al., 2020). The VR system was installed in an empty room of similar size to that of the Virtual Shop so that participants could actually walk, explore, and select objects in the environment as seen through an audio-visual Head Mounted Display (HMD). The Virtual Shop was run by the program 3DVIA Virtools 5 on a Dell Precision T3600 PC with an Inter(R) Xeon (R) CPU ES-1620 0 (3.60 Ghz, 10 Gbytes in RAM) processor and a NVIDIA GeForce GTX 600 Ti graphics card. Three-dimensional visual images were presented using the HMD nVisor ST50. The HMD provided stereoscopic vision *via* two screens placed in front of the eyes (1,280 × 1,024 full color with 50°diagonal field-of-view) and sound by stereo headphones. The HMD was connected to a PPT-X system (6°of freedom) motion tracker by WorldViz, which transmits head position/rotation to a Shuttle PC computer to provide real-time updating of the virtual environment. It allows the user to rotate his/her head in a 360°view and to walk freely in the virtual environment. A motion tracker was also attached to the participant’s right hand. The participant moved his/her hand to position a red circle on the objects they wanted to select. A handheld device was used to point and select virtual objects. When immersed in the environment, participants who were allocated to the VR+ condition were asked to complete the following exercises: The participants were first positioned in front of the store counter behind which a virtual cashier was standing. The experimenter explained that a virtual notepad located on the countertop would show a list of six images of objects (e.g., milk, candles, etc.) and that they should memorize these objects, as they would later have to find and select them in the virtual store. After these six images were presented, the virtual cashier engaged in a conversation with the participant to create interference by asking a set of brief questions (e.g., “What is the weather like today?”). After 20 s, the cashier instructed the participant to walk into the store to retrieve the objects from the list. The six objects were randomly placed in different locations in the store. The store also contained six distractor objects, which belonged to the same semantic category as the learned objects. The participants were free to explore the store as they wished and there was no time limit to retrieve the objects. They were also informed that they could use the method of loci if they wanted. Participants allocated to the VR− condition were presented the same objects as the VR+ condition and were asked to find the objects in the store. The main difference was that only one object was presented at a time in the VR− condition and it remained visible until it was found and selected. As the object remained visible, the procedure did not involve memory as only one object was presented at a time. The procedure was repeated for the six objects from the list. Thus, the visual and motor component of the VR− and VR+ conditions were similar. The VR− active control condition controlled for exposure to the Virtual Shop environment, objects, and characters, and the manipulation of the device used to select objects. This was critical to ensure that improvement on the transfer task observed in the VR+ group was not due to increased practice in the VR environment.

### Outcomes Measures

#### Measure of Efficacy

A word recall task was used to measure training efficacy. Participants were asked to memorize two lists of 12 words. Items were presented auditorily at a rate of one item every 5 s and recall was done in writing. Words were presented using the E-prime 2 software with Plantronix Audio 550 headphones. The mean number of words correctly recalled was used as the dependent variable.

#### Measures of Context Transfer

##### The Virtual Shop

A first immersive VR memory task was the *Virtual Shop*, a task reported as a feasible and valid measure of memory in older adults (Ouellet et al., 2018; Lecavalier et al., 2020). The participant was immersed in a Virtual Shop environment using a physical layout that differed from the one used during training. The physical appearance of the shops used in the training and testing phases differed in terms of the placement and orientation of the shelves as well as the colors and material of the shelves, floor, and walls. While in the shop, the participant was asked to memorize, find, and select 12 objects. Objects were first presented on a notepad like the one used in the training environment at a rate of one object every 5 s. This was followed by a 20-s conversation with the cashier, where he asked questions regarding the soccer game seen on TV and the time indicated on the clock. Following the interference period, participants were asked to find the learned objects in the shop and select each of them with a remote control. The learned objects were placed randomly in the store among 12 distractor objects, which belonged to the same semantic categories as the target items. Encoding and retrieval were done in a noisy environment: participants heard conversations, which consisted of two short texts read respectively by male and a female and presented dichotically through the HMD headphones. The number of learned objects correctly recalled (i.e., selected and validated) was used as the dependent variable.

##### The Virtual Car Ride

A second immersive VR task was a modified version of the one used in prior work to assess transfer after attentional training in older participants (Bier et al., 2018). The participant was immersed in a 3D virtual car, where he/she was a passenger giving the driver directions. The virtual car was driven on a highway, and road signs with directions to Chauminont, along with other city names, were shown along the way. The participant was instructed to detect the road signs with directions for the city of Chauminont by pressing a button. Using their left index finger, participants pressed the left mouse button each time they saw the direction for Chauminont on the road signs. A recording of a radio station broadcast about traffic reports was used as verbal noise during the task. Concurrently, a list of 12 concrete words was read by a male voice at a rate of one word every 5 s. The participant was asked to encode and verbally recall the words immediately after presentation of the list, while identifying the Chauminont road signs. The Virtual Car Ride ended when the participant finished reading the words. Thus, it lasted about 4 min and included a maximum of 40 road signs (half of them targets and half distractors). Memory performance (i.e., the number of words correctly recalled, and accuracy in detecting road signs [(hits minus false alarms)/number of targets presented] served as dependent variables.

#### The Multifactorial Memory Questionnaire

The multifactorial memory questionnaire (MMQ) is a well-validated self-report questionnaire measuring memory in everyday life (Troyer and Rich, 2002). The French version of the MMQ was used in the present study (Fort et al., 2004). It comprises three subscales: The contentment scale is a measure of affects regarding memory; the strategy subscale measures the different strategies that people use in their daily life; and the ability subscale measures the impact of memory problems in daily life. Here, we focused on the ability subscale, as the two other subscales reflect components that were not considered sensitive to the type of training provided here. The ability subscale comprised 20 items where participants rated how often they encountered memory difficulties in their daily life (e.g., How often do you forget to pay a bill on time? How often do you misplace something you use daily like your keys or glasses?, etc.) over the last 2 weeks, on a scale from *all the time* (1) to *never* (5). The internal consistency of the French version of this scale is highly reliable and measured by Cronbach’s alpha, *α* = 0.88 (Fort et al., 2004).

### Statistical Analyses

All data were analyzed using the *Statistical Package for Social Sciences* (SPSS) version 21.0. Independent *t*-tests (two-tailed) and chi-square tests were used to evaluate between-group differences at baseline. To assess efficacy on the word recall task and transfer on the Virtual Shop and the Virtual Car Ride, separate mixed ANOVA were conducted with condition (VR+; VR−) as a between-subject factor and phase (PRE; POST 3; POST 6) as a within-subject factor. Transfer measured with the ability score from the MMQ was analyzed with condition (VR+; VR−) as a between-subject factor and phase (PRE; POST 6) as a within-subject factor. Paired comparisons were then conducted for *post hoc* analyses. A main phase effect was expected for efficacy on word recall, as all participants were expected to improve. A phase effect was expected on context transfer measures in both groups, as mild transfer effects were expected to occur. However, the phase effect should be qualified by a phase × condition interaction to reflect greater transfer in the VR+ than VR− condition. *Pearson* bivariate correlations (one-tailed) were computed to assess the relationship between change scores on measures of efficacy and measures of transfer at POST 3 and POST 6. Correlations were used to assess whether improvement on transfer measures reflect training efficacy. A standard α-level of 0.05 was used for all analyses.

## Results

### Baseline Characteristics and Pre-training Performance

**Figure 3:**
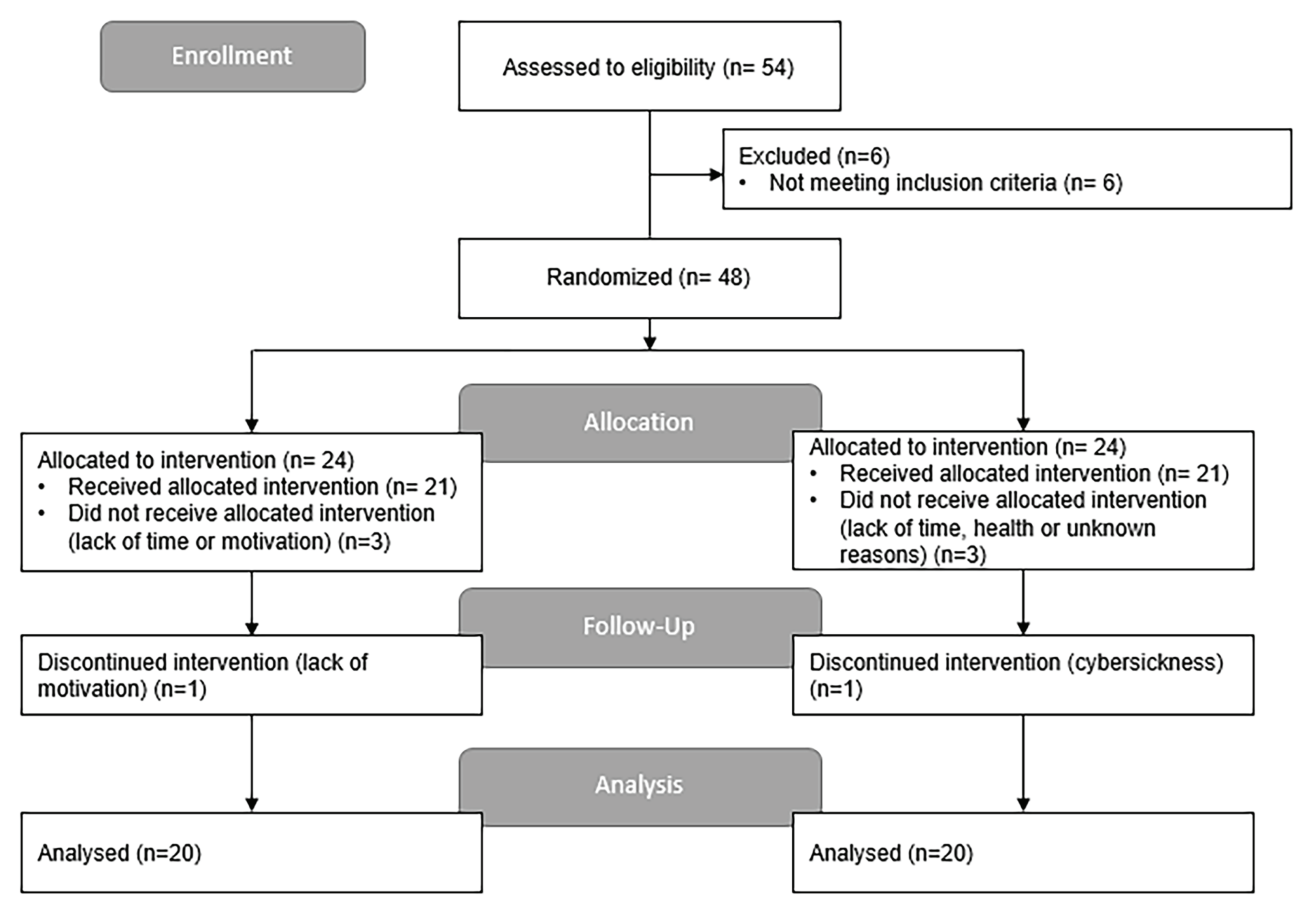
Flow chart of participants.

Figure 3 shows the participant flow in the study. Participants were recruited between 2013 and 2015 and testing was conducted from September 2014 to June 2015. Out of the 54 participants assessed for eligibility, six were excluded based on impaired memory performance in neurological tests. The 48 remaining participants were then randomized. Six participants did not receive the allocated intervention due to lack of time or motivation, health-related or unknown reasons and were thus excluded from the analysis. Two other participants were also excluded from the analysis as they discontinued the intervention (see Figure 3). Data from the 40 remaining participants (VR+, *n* = 20; VR−, *n* = 20) were analyzed. The baseline characteristics of the participants who completed the intervention are presented below in Table 1. The groups did not differ on any of the variables at baseline.

**Table 1:** Baseline characteristics of participants randomized to the VR+ and VR− training conditions.

	VR+ (*n* = 20)	VR− (*n* = 20)
Gender (Women/Men)	16/4	17/3
Age	66.00 (7.67)	68.60 (7.27)
Education	15.30 (2.87)	14.70 (3.37)
ADL-PI (/30)	2.15 (2.83)	0.75 (1.48)
GDS (/15)	2.35 (3.79)	1.45 (1.76)
MoCA (/30)	27.80 (1.54)	27.50 (1.73)
Hachinski scale (/18)	1.23 (0.28)	0.75 (0.17)
Charlson comorbidity index (/41)	0.55 (0.76)	0.55 (0.83)
Boston naming test (/15)	13.90 (1.17)	13.80 (1.28)
RL/RI delayed free recall (/16)	12.55 (1.93)	11.55 (1.36)
LM I (immediate recall) (/25)	15.30 (3.92)	14.70 (4.49)
LM I (delayed recall) (/25)	14.35 (4.40)	14.00 (3.23)
Stroop (time on third plate)	28.26 (8.35)	28.84 (6.31)
Vocabulary (scale score, 1–19)	14.00 (2.20)	13.65 (2.41)

### Measure of Training Efficacy

**Figure 4:**
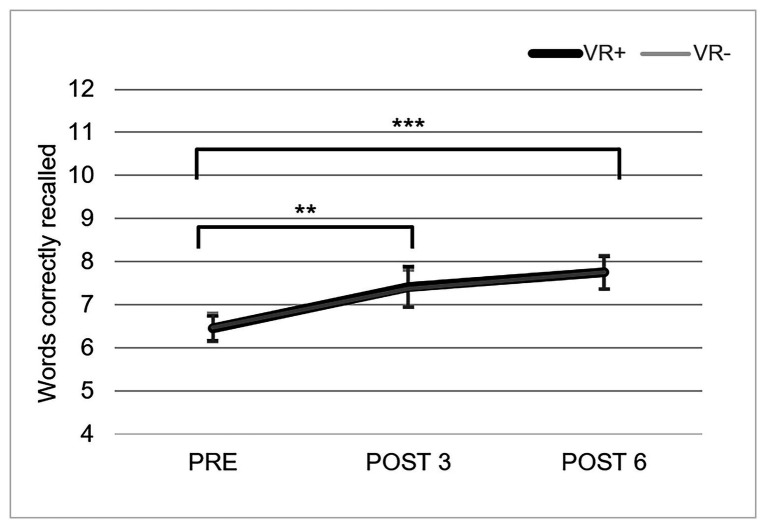
Performance on the measure of training efficacy. The mean of words correctly recalled on measure of training efficacy for the three testing phases in the VR+ (black) and VR− (gray) training conditions. Error bars represent SE. ^**^p < 0.01 and ^***^p < 0.001. Paired comparisons indicated a significant difference between PRE and POST 3, and between PRE and POST 6.

The mean number of words correctly recalled in the two training conditions over the three testing phases is presented in Figure 4. The condition × phase ANOVA indicated a main effect of phase, *F*(2,37) = 12.55, *p* = 0.00002; *η*^2^_p_ = 0.25. Paired comparisons indicated that performance increased from PRE to POST 3 (*p* < 0.01) and that there was no further improvement from POST 3 to POST 6. The condition *F*(2,37) = 0.0003, *p* = 0.99 and condition × phase interaction, *F*(2,37) = 0.01, *p* = 0.99, were not significant.

### Transfer Measures

#### The Virtual Shop

**Figure 5:**
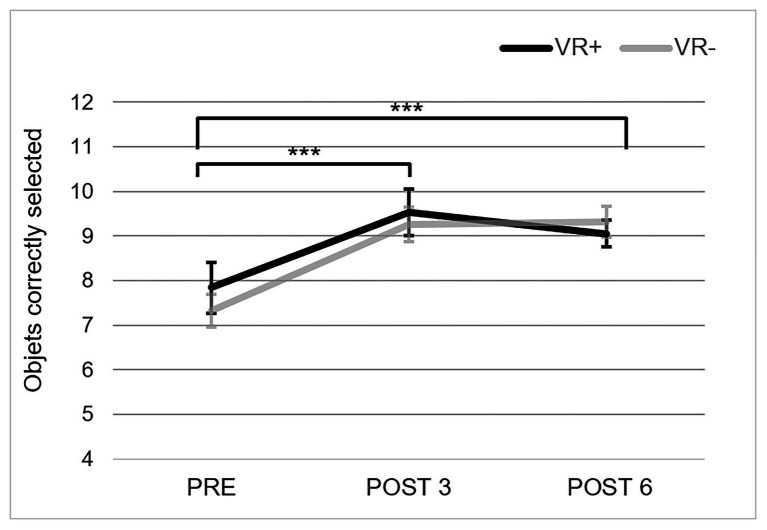
Performance in the Virtual Shop. Correct recall in the Virtual Shop for the three testing phases in the VR+ (black) and VR− (gray) training conditions. ^***^p < 0.001, paired comparisons showed significant difference between PRE and POST 3, and between PRE and POST 6.

The mean number of objects correctly retrieved in the virtual environment for the three testing phases and the two training conditions is presented in Figure 5.^3^ The ANOVA indicated a phase effect, *F*(2,35) = 13.73, *p* = 0.00001; *η*^2^_p_ = 0.28. Paired comparisons revealed that this was due to a significant improvement from PRE to POST 3 (*p* < 0.001) with no further improvement from POST 3 to POST 6. There was no effect of condition, *F*(2,35) = 0.18, *p* = 0.68 or condition × phase interaction, *F*(2,35) = 0.56, *p* = 0.57.

#### The Virtual Car Ride

**Figure 6:**
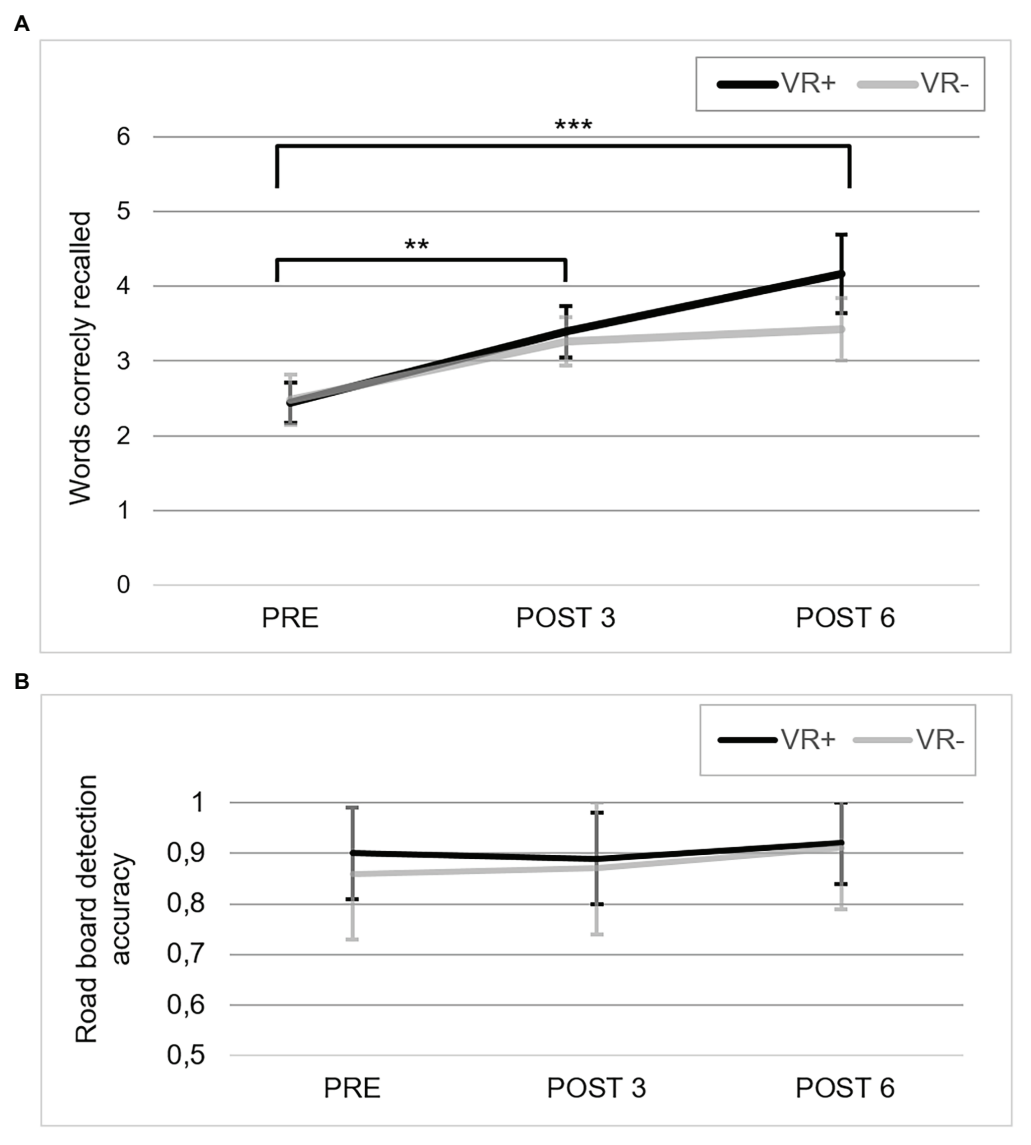
Performance in the Virtual Car Ride. **(A)** Correct recall in the Virtual Car Ride for the three testing phases in the VR+ (black) and VR− (gray) training conditions. ^**^p < 0.01 and ^***^p < 0.001, paired comparisons showed significant differences between PRE and POST 3, and between PRE and POST 6. **(B)** Proportion of accuracy for the three testing phases for road board detection in the VR+ (black) and VR− (gray) training conditions.

Performance on the Virtual Car Ride task over the three testing phases and the two training conditions are presented in Figure 6A for word recall and Figure 6B for road sign detection.^4^ The ANOVA on word recall indicated a main effect of Phase, *F*(2,34) = 8.35, *p* = 0.001; *η*^2^_p_ = 0.19. Paired comparisons revealed that this was due to a significant improvement from PRE to POST 3, *p* < 0.05, with no further improvement from POST 3 to POST 6. The ANOVA on road sign detection revealed no significant effect of training condition, *F*(2,34) = 0.05, *p* = 0.83, Phase, *F*(2,34) = 1.62, *p* = 0.21, or interaction *F*(2,34) = 0.51, *p* = 0.58.

#### The MMQ Questionnaire

**Figure 7:**
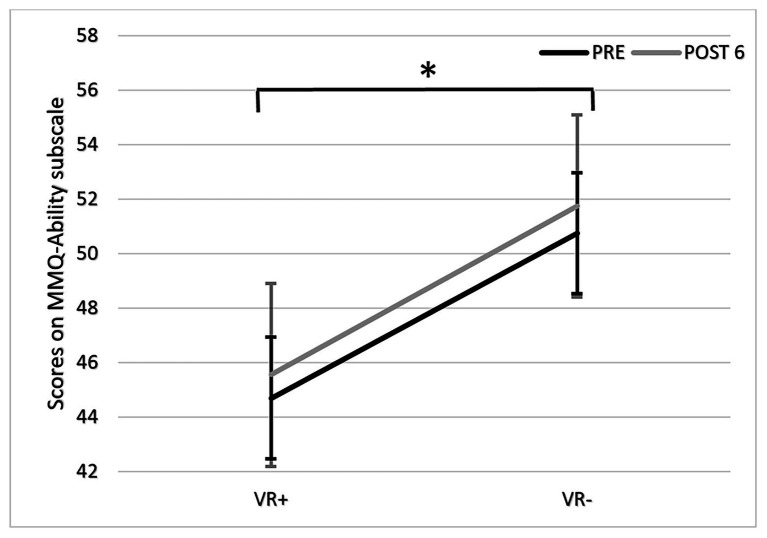
Scores on multifactorial memory questionnaire (MMQ)-Ability subscale at PRE (black) and POST 6 (gray) in the VR+ and VR− training conditions. ^*^p < 0.05, paired comparisons showed significant differences between VR+ and VR− conditions.

Scores on MMQ-Ability subscale at PRE and POST 6 for both VR+ and VR− groups are presented in Figure 7.^5^ The ANOVA revealed no significant effect of phase, *F*(1,37) = 1.43, *p* = 0.24, or interaction, *F*(1,37) = 0.41, *p* = 0.53 but there was a main effect of condition, *F*(1,37) = 6.11, *p* = 0.018, *η*^2^_p_ = 0.14 due to the VR+ group showing lower scores than the VR− group overall.

### Relationship Between Training Efficacy and Transfer Measures

A significant correlation was found between improvement on word recall and improvement on the Virtual Car Ride (*r* = 0.32, *p* < 0.05). The positive correlation indicates that a larger improvement on the efficacy measure is associated with a larger improvement in the Virtual Car Ride. None of the other correlations were significant.

## Discussion

The first goal of this study was to assess whether VR can be used to measure real-life transfer of strategic memory training. The second goal was to determine if transfer is increased when training is practiced in a VR environment replicating real-life conditions where the learned strategy can be applied. Older adults with memory complaints were randomized to two memory training conditions, which included (VR+) or did not include (VR−) memory exercises in VR. Participants were assessed before training (PRE), and after three (POST 3) and six (POST 6) training sessions with one measure of training efficacy, three measures of *context transfer*, two VR tasks and one self-report scale. Overall, results confirm prior results indicating that strategic memory training is effective to increase performance on word recall. Improvements were also found on the two VR memory tasks but not on the memory questionnaire. Interestingly, improvement on word recall and VR memory tasks was found when comparing PRE to POST 3, with no further improvement at POST 6. However, there was no advantage of coupling the memory training with VR memory exercises. Thus, we found no evidence supporting an increase in transfer effects when enriching training with VR memory exercises that mimic real-world situations.

### Measuring Context Transfer

One innovative aspect of this study resides in the utilization of VR to evaluate context transfer, with the assumption that VR is more likely to capture different dimensions of real-life transfer than what is reflected by self-report questionnaires. Thus, in addition to a self-report questionnaire, two measures were used to assess context transfer: a version of the Virtual Shop that was slightly different from the one used in the VR+ group and the Virtual Car Ride, where participants recalled words while multi-tasking. The results showed better recall in the VR environments from PRE to POST 3, on both the Virtual Shop and Virtual Car Ride, but no transfer on the self-report questionnaire. Interestingly, the two VR context transfer tasks are different in terms of their proximities to the training conditions. The Virtual Shop is close to the shopping scenario used during training in both conditions, with similar input and response modalities. The Virtual Shop can be considered a near-context transfer task, as the version of the shopping environment was not similar to the one used during VR exercises and the items to memorize were twice as numerous as in the VR exercises. In turn, the Virtual Car Ride places participants in a context that is quite different from the training conditions. Yet, both tasks improved following training, which suggests presence of transfer. It is important to stress that in the absence of a no-contact group, we cannot reject that improvement can be the result of a practice effect and fully support generalization to contexts other than that of the memory training condition (Rebok et al., 2007; Brehmer et al., 2014; Kelly et al., 2014; Melby-Lervåg et al., 2016). Note that we found a positive correlation between improvement on word recall and the Virtual Car Ride, but not the Virtual Shop. This partly supports an interpretation that the PRE-POST improvement on the Virtual Car Ride reflects transfer rather than merely practice effect.

The absence of change on the MMQ Ability subscale score following training is consistent with prior work indicating small effects on self-report measurements (Chandler et al., 2016). Consistent with our findings, Bier et al. (2018) reported improved dual-task performance in a real-life VR scenario following a variable priority attentional training in older adults, but no impact on a self-report questionnaire. The MMQ was chosen for its ability to reflect the self-perception of encountering memory difficulties in daily life over the previous 2 weeks. We hoped that the method of loci could be used in some situations where memory is required in daily life. However, the MMQ includes questions about where the learned strategy would not have been useful, and thus, the subscale was not perfectly adapted to the learned strategy. Therefore, future studies may benefit from developing self-report questionnaires that better reflect situations where strategies can be applied. Alternative or complementary reasons may explain the inability of self-report questionnaires to reflect context transfer. First, it has been shown that responses on self-report questionnaires are more influenced by mood (Derouesné et al., 1999) and personality traits (Barker et al., 1995) than objective memory performance. Second, subjective measures reflect the global judgment of one’s own memory based on the general knowledge that this person has about his/her memory (metacognitive knowledge). This general knowledge reflects a person’s representation of his/her capacity based on a multitude of memory experiences and as a result metacognition to be stable over time (McDonald-Miszczak et al., 1995; Weaver Cargin et al., 2008). As a result, subjective complaint may be relatively insensitive to change, as it probably requires many instances of “positive” or “improved” memory experiences to be modified and decline in meta-memory accuracy. This has been shown in populations known to have memory complaints, such as in those with MCI (Perrotin et al., 2007).

Our finding suggests that VR has potential as a measure of transfer, although the design of the study does not allow the rejection of the hypothesis of a practice effect. This is consistent with previous studies showing that VR assessments can predict real-world performance (Renison et al., 2012; Allain et al., 2014), contrary to traditional experimental tasks (Chaytor and Schmitter-Edgecombe, 2003). This does not come as a surprise as experimental memory tasks are often word lists, which are very different than memorizing in challenging everyday situations, where an individual may be dual-tasking while navigating or in noisy environments, etc. Interestingly, previous studies have shown that VR cognitive assessments have the further advantage of being more motivating than traditional cognitive tasks for older adults and generate a good sense of “presence” in this population (i.e., being fully attentive and immersed in the task; Lecavalier et al., 2020), two variables associated with memory performance (Hess et al., 2012; Lecavalier et al., 2020).

### The Role of VR in Increasing Training Context Transfer

A second goal was to test if practicing the memory strategy in a complex VR environment increased *context transfer*. Contrary to our expectations, the VR+ training group did not show better performance on the efficacy measure or improved *context transfer* compared to the VR− training group.

This is counter to the few studies showing that full VR memory training (Man et al., 2012) or cognitive intervention, including VR exercises (Optale et al., 2010), resulted in greater cognitive improvement than non-VR training. One important difference between the methods used in these studies and the present one is the shorter duration of the virtual immersion here, which aims to reduce the likelihood of cybersickness symptoms. Many studies have reported nausea, headaches, and disorientation with long-duration immersion in virtual environments (e.g., Jaeger and Mourant, 2001). In a previous study, assessing applicability of the Virtual Shop to healthy older adults, a very low level of cybersickness was detected for a completion time of less than 13 min (Lecavalier et al., 2020). Thus, to reduce the risk of cybersickness symptoms, we provided relatively short practice sessions in the virtual environment. This is contrary to Optale’s and Man’s study, both of which allocated 30 min to the VR experience. This could have reduced the likelihood of observing an effect due to VR training. Thus, insufficient training dosage might explain our failure to observe an impact of VR training on efficacy or transfer. There are other reasons that may explain why we did not find a larger transfer in the VR+ condition. One reason is that the VR training exercises were not appropriate, i.e., not specific, complex, or rich enough, to impact cognition beyond the traditional method of loci learning. Another reason is that the length of the immersions or the number sessions provided in VR may have been insufficient to yield transfer. Another possible methodological reason is our lack of a no-contact control condition. It is possible that the active control condition used here (VR−) was sufficient to produce transfer.

Our design was built to assess transfer of the training efficacy in close to real-life situations. VR exercises were implemented to assess whether practicing the method of loci in these situations could boost or amplify transfer. The VR tasks had to be amenable to using the method of loci, such as memorizing a list of items to purchase. For this reason, the VR exercises used in the VR+ condition were closed to the exercises used to assess transfer, as both involved memorizing and retrieving the list of items. However, these two versions of the exercises differ on two points: environment and task difficulty. First, the VR environments were visually different for the two versions, i.e., the floor and wall coverings, and the sets of items. Second, the number of items to remember in the Virtual Shop (*n* = 12) were twice as numerous as those used in the VR exercises (*n* = 6). Notwithstanding a possible practice effect, these differences were introduced to reduce their impact. Furthermore, it should be noted that one of the transfer tasks, the Virtual Car Ride, was quite dissimilar to the trained task. Since this task also improved after training, our effects are unlikely to be related to a mere practice effect.

Lastly, it is possible that the effect size for the VR+/VR− comparison was smaller than the one calculated for training efficacy and hence requires a larger sample size to detect a significant difference. However, the challenge of finding transfer is well recognized, and it is unlikely that a straightforward explanation exists, as large scale studies (e.g., Ball et al., 2002) have failed to find transfer in memory training. Hence, our approach was to design an intervention that would directly integrate strategies favorable to transfer. Undoubtedly, more research is needed to identify the optimal conditions for VR training transfer.

### The Effect of Training Dose

The design included three measurements to assess the effect of cumulative training dose on efficacy and transfer. One of our motivations to assess training dose was to determine whether limited transfer is due to insufficient training dosage. In addition, there could have been a lag between the occurrence of efficacy and transfer, where transfer effects may require a larger dose than efficacy. In both cases, changes were found to occur from PRE to midway through training with no further improvement afterward. This pattern is compelling as it was found on all measures. This is consistent with previous findings indicating that increasing training dose has a relatively small cumulative effect on training gains when using strategic training, contrary to training that involves repeated practice where cumulative dose is important (for meta-analyses, see Gross et al., 2012; Schwaighofer et al., 2015). Furthermore, the fact that the training effect occurred quite rapidly (i.e., after only three sessions of training) is consistent with prior studies showing that a single session with the method of loci preceded by a session on training mental imagery was sufficient for most participants to learn to use the method (Belleville et al., 2006, 2018). The results that we obtained and the meta-analysis from Gross et al. (2012) suggest that improvement in strategic learning might follow a non-linear pattern: performance may improve rapidly at the beginning of the training as people learn the strategy, but with no further effect with increased dose once the strategy is mastered. In the present study, we used three additional sessions to consolidate learning, as we figured it may help maintain performance and perhaps favor transfer. However, one interesting implication of such a rapid effect is that the benefit from training on the method of loci can be observed following a relatively low dose, although this needs to be confirmed. If confirmed, this is important because clinical environments have often limited financial and human resources, which prevents the implementation of long-term intervention programs. Note that while increasing training dose has no short-term impact, we cannot exclude that overlearning facilitates long-term maintenance of transfer gains.

### Limitations

It is important to address some of the limitations of the study. First, we did not include a no-contact condition, which makes the interpretation of our finding more complex because we cannot rule out that some of the effect comes from a test-retest effect rather than reflecting transfer. However, finding a positive correlation between change scores on the measure of training efficacy and change scores on transfer suggests that this improvement is due to efficient learning and use of the method of loci. Additional studies are needed to better explore the transfer effects into real-life outcomes such as functionality. Measuring real-life with functionality is challenging because it relies mostly on self-reported questionnaires, which have limitations (Ball et al., 2002; Willis et al., 2006; Rebok et al., 2014; Belleville et al., 2018). Our hypothesis was that using VR measures would help circumvent these limitations by having participants perform tasks that are close to real-life conditions. The advantage of VR as a measure of cognition in real-life is exemplified by Bier et al. (2018), who report evidence of transfer after attentional training on the Virtual Car Ride but not the self-reported questionnaire. Undoubtedly, one area of future research will be to better examine the relationship between VR real life tasks and self-report questionnaires. Another limitation of the present study is related to cybersickness symptoms. Despite a relatively short duration, some participants experienced cybersickness symptoms during VR exercises (*n* = 1) or VR transfer tasks (*n* = 3). It is possible that participants walking around the room contributed more to symptoms compared to studies where they navigated using a joystick, while sitting on a chair (e.g., Optale et al., 2010). This suggests that these symptoms will have to be considered in future studies or when establishing the clinical utility of VR. Interestingly, there are more recent systems with better optical properties, which will likely reduce the presence of cybersickness symptoms and therefore improve usability of the technology. Lastly, the Virtual Shop and the Virtual Car Ride were constructed to be closer to real-life memory situations – and thus more ecologically valid – than traditional experimental tasks. Yet they remain experimental procedures as the task is relatively confined in space and time, participants are instructed about the goals of the task, and the encoding and retrieval phases are not entirely self-initiated. The VR tasks also require interaction with technological material (wearing an HMD, using a remote or mouse, etc.), and the performance of older adults may be influenced by their degree of familiarity with new technologies (Iverson et al., 2009; Lopez et al., 2016). Note however, that evidence from earlier work suggests that VR tasks are feasible and valid in older adults (Ouellet et al., 2018; Lecavalier et al., 2020).

## Conclusion

In summary, our findings indicate that providing a low dose of strategic memory training to older adults with subjective complaints leads to increased performance on VR memory tasks, which were used here as measures of transfer. The results confirm that memory training based on strategies, such as the method of loci, is a promising non-pharmacological approach that can help seniors with memory complaints cope with everyday memory difficulties. Furthermore, as these individuals are susceptible to being in a prodromal phase of Alzheimer’s disease (Jessen et al., 2014), providing memory training may delay the impact of the disease on functional autonomy. VR proved to be potentially useful tool to measure transfer effects in situations that are close to daily life in this population. However, when attempting to enhance the ecological validity of the training by adding VR memory exercises to a classical memory training, we failed to find that this provided added benefit. Though promising, more research is needed in to demonstrate the usefulness and feasibility of using VR in general clinical situations.

## Data Availability Statement

The raw data supporting the conclusions of this article will be made available by the authors, without undue reservation.

## Ethics Statement

The studies involving human participants were reviewed and approved by Comité d’éthique de la recherche vieillissement-neuroimagerie (CER VN) and Centre intégré universitaire de santé et de services sociaux du Centre-Sud-de-l’Île-de-Montréal. The patients/participants provided their written informed consent to participate in this study.

## Author Contributions

BB, ÉO, and SB conceived and designed the study, contributed to the first draft of the manuscript, and analyzed the data. BB and ÉO performed the experiments and created the visualizations. SB verified the analyses, supervised the study, and provided resources and financial support. BB conducted the research process. BB and SB contributed to the final version of the manuscript. All authors contributed to the article and approved the submitted version.

### Conflict of Interest

The authors declare that the research was conducted in the absence of any commercial or financial relationships that could be construed as a potential conflict of interest.

## Footnotes

^1^The self-report questionnaire was completed only at PRE and POST 6. The questionnaire refers to the past 2 weeks and hence, the time period covered by POST 3 would overlap with that of PRE.

^2^The training protocol also included an attentional training received by half of the participants in each VR group. The attentional training did not have any effect on either efficacy or transfer measures and hence will not be further discussed here for the sake of simplicity.

^3^Note that two participants (one from VR+, one from VR−) did not complete the Virtual Shop assessment due to cybersickness symptoms. Consequently, the following analyses were conducted on 38 participants.

^4^Note that three participants (two from VR+, one from VR−) did not complete the Virtual Car Ride assessment due to cybersickness symptoms. Consequently, the following analyses were conducted on 37 participants.

^5^Note that one participant (from VR−) was excluded as he/she did not complete all questions from the questionnaire. Consequently, the following analyses were conducted on 39 participants.
